# Population structure of *Streptococcus oralis*

**DOI:** 10.1099/mic.0.027284-0

**Published:** 2009-08

**Authors:** Thuy Do, Keith A. Jolley, Martin C. J. Maiden, Steven C. Gilbert, Douglas Clark, William G. Wade, David Beighton

**Affiliations:** 1 King's College London Dental Institute at Guy's, King's College and St Thomas' Hospitals, Infection Research Group, Guy's Campus, London SE1 9RT, UK; 2 Biomedical Research Centre, Guy's and St Thomas' Hospital NHS Foundation Trust, London SE1 9RT, UK; 3 Department of Zoology, University of Oxford, Oxford OX1 3PS, UK

## Abstract

*Streptococcus oralis* is a member of the normal human oral microbiota, capable of opportunistic pathogenicity; like related oral streptococci, it exhibits appreciable phenotypic and genetic variation. A multilocus sequence typing (MLST) scheme for *S. oralis* was developed and the resultant data analysed to examine the population structure of the species. Analysis of 113 isolates, confirmed as belonging to the *S. oralis/mitis* group by 16S rRNA gene sequencing, characterized the population as highly diverse and undergoing inter- and intra-species recombination with a probable clonal complex structure. ClonalFrame analysis of these *S. oralis* isolates along with examples of *Streptococcus pneumoniae*, *Streptococcus mitis* and *Streptococcus pseudopneumoniae* grouped the named species into distinct, coherent populations and did not support the clustering of *S. pseudopneumoniae* with *S. mitis* as reported previously using distance-based methods. Analysis of the individual loci suggested that this discrepancy was due to the possible hybrid nature of *S. pseudopneumoniae*. The data are available on the public MLST website (http://pubmlst.org/soralis/).

## INTRODUCTION

The viridans streptococci form a major component of the oral and pharyngeal microbiota. They can be categorized into a number of phylogenetic groupings, one of which, the mitis group, includes the common oral species *Streptococcus mitis* and *Streptococcus oralis* as well as the pathogen *Streptococcus pneumoniae*. *S. pneumoniae* is a major cause of both local and systemic infections, and several of the other mitis group streptococci, particularly *S. oralis* and *S. mitis*, are recognized as important aetiological agents of subacute bacterial endocarditis and septicaemia in neutropenic cancer patients (Paddick *et al.*, 2003; Whiley & Beighton, 1998; Wisplinghoff *et al.*, 1999). *S. pneumoniae*, *S. pseudopneumoniae*, *S. mitis* and *S. oralis* are closely related phylogenetically; they exhibit >99 % 16S rRNA sequence identity and are difficult to differentiate using conventional biochemical tests (Arbique *et al.*, 2004; Whatmore *et al.*, 2000).

To improve the reliability of the identification of these four species, analysis of the nucleotide sequences of single housekeeping genes has been suggested, and genes encoding d-alanine–d-alanine ligase (*ddl*) (Garnier *et al.*, 1997; Kawamura *et al.*, 1999; Okwumabua *et al.*, 2003), the *β* subunit of RNA polymerase (*rpoB*) (Drancourt *et al.*, 2004), manganese-dependent superoxide dismutase (*sodA*) (Kawamura *et al.*, 1999; Poyart *et al.*, 1998, 2002), *groES* and *groEL* (Teng *et al.*, 2002) have been used. Phylogenetic analysis of streptococcal isolates using concatenated sequences of housekeeping genes indicated that isolates identified as *S. pneumoniae* formed a discrete group, *S. oralis* isolates formed two further groups and *S. mitis* isolates did not cluster into a well-separated group (Whatmore *et al.*, 2000). More recently, Hoshino *et al.* (2005) amplified and sequenced internal regions of each of four housekeeping genes (*ddl, gdh, rpoB* and *sodA*). The phylogenetic analyses based on these individual genes did not differentiate all strains of *S. pneumoniae*, *S. pseudopneumoniae*, *S. mitis* and *S. oralis*. The gene fragments used in the multilocus sequence typing (MLST) scheme of *S. pneumoniae* (*aroE*, *ddl*, *gdh*, *gki*, *recP*, *xpt*, *spi*) have also been determined for a range of clinical isolates of *S. pneumoniae*, *S. mitis* and *S. oralis*, and analysis of sequences when concatenated enabled strains of *S. pneumoniae*, *S. mitis* and *S. oralis* to be identified, with both *S. oralis* and *S. mitis* exhibiting extensive intra-species diversity (Chi *et al.*, 2007).

The phylogenetic relationships among *S. pneumoniae*, *S. mitis*, *S. pseudopneumoniae* and *S. oralis* seemed to be clearly established but it has been recently suggested that *S. pneumoniae* is the ancestor of *S. mitis*, *S. pseudopneumoniae*, *Streptococcus infantis* and *S. oralis*, with the latter species having lost the virulence determinants still present in *S. pneumoniae* (Kilian *et al.*, 2008). Phylogenetic trees constructed from nucleotide sequences of the *ddl*, *gdh*, *rpoB* and *sodA* genes of *S. pneumoniae*, *S. mitis*, *S. infantis* and *S. pseudopneumoniae* have been interpreted as containing several hundred evolutionary lineages, one of which is the virulent species *S. pneumoniae*. In this framework, it is proposed that the commensal species *S. mitis* is represented by multiple lineages, each of which represents a separate species by traditional taxonomic standards, and that *S. pseudopneumoniae* is one of these discrete lineages; however, the population structure of *S. oralis* was not investigated, except to demonstrate that the species was distinct from the others (Kilian *et al.*, 2008).

*S. oralis* is a numerically important member of the commensal oral microbiota, isolated from all intra-oral surfaces and a pioneer organism involved in the primary colonization of the dentition (Nyvad & Kilian, 1990). Genotyping studies using repetitive extragenic palindromic (REP)-PCR have shown that *S. oralis* is usually present as multiple genotypes in the same individual and that it is rare for unrelated individuals to share the same genotypes (Alam *et al.*, 1999; O'Neill *et al.*, 1999). Extensive sequencing of *gdh* alleles of members of the ‘oralis-pneumoniae-mitis’ group in samples from two subjects found that the sequences clustered with the previously described species (Bek-Thomsen *et al.*, 2008). However, these data did not provide an understanding of the population structure of *S. oralis*. In the present study a collection of isolates identified on the basis of 16S rRNA sequences as belonging to the ‘oralis-pneumoniae-mitis’ group of streptococci was investigated. The isolates were genotyped with REP-PCR, and an MLST-based approach was employed to investigate their population structure and phylogenetic relationships to closely related species.

## METHODS

### Streptococcal isolates.

The streptococci analysed (*n*=113) were isolated from healthy or diseased sites in a range of subjects (*n*=57) in the UK between 1985 and 2006 (see Supplementary Table S1, available with the online version of this paper). Briefly, 72 isolates were from infected dentine of active root caries lesions, 1 from subgingival dental plaque, 12 from interproximal caries-free sites, 9 from infected dentine of occlusal carious lesions, 16 from the supragingival plaque of caries-free subjects and 3 from dentoalveolar abscesses. To confirm these presumptive identities the isolates were grown overnight at 37 °C on Columbia agar (Oxoid) supplemented with 5 % (v/v) defibrinated horse blood, anaerobically (MACS-MG-1000 anaerobic workstation, Don Whitley Scientific). DNA was extracted, and amplification and sequencing of 16S rRNA was carried out using the universal primers 27f and 1492r for amplification and 519r for sequencing (Lane, 1991). These sequences were subjected to blast searching (http://www.ncbi.nlm.nih.gov/blast/Blast.cgi) and also submitted to the Ribosomal Database Project (Wang *et al.*, 2007) (http://rdp.cme.msu.edu/) to confirm that isolates exhibited >98 % identity with the 16S rRNA sequences of the type strains of *S. oralis* or *S. mitis*. Single independent isolates were studied to determine variability between subjects, and additionally multiple isolates from a number of subjects were examined in order to investigate the variability of isolates within the same subject and between subjects. The type strains of *S. oralis* (NCTC 11427), *S. mitis* (NCTC 12261) and *S. pseudopneumoniae* (DMZ 18670) were also studied. All isolates were preserved in vials containing plastic beads in cryopreservative fluid (Technical Service Consultants Ltd) and stored at –80 °C.

### REP-PCR analysis.

REP-PCR patterns were determined for all isolates using the previously reported method (Alam *et al.*, 1999). All of the patterns were compared using GelCompar version 4.0 (Applied Maths). The individual bands in each of the patterns produced by the different PCR methods were analysed by applying the Dice coefficient. For clustering, the unweighted pair group method using mathematical averages (UPGMA) was used, and a band position tolerance of 1.5 % applied. On the basis of previous analyses, isolates were considered identical if they exhibited >95 % similarity.

### Choice of loci for identification.

The loci used for typing *S. oralis* and *S. mitis* were based on those used in the MLST scheme of *S. pneumoniae* (Enright & Spratt, 1998) (http://spneumoniae.mlst.net). The genes were *aroE* (shikimate dehydrogenase), *ddl* (d-alanine–d-alanine ligase), *gdh* (glucose-6-phosphate dehydrogenase), *gki* (glucokinase), *recP* (transketolase) and *xpt* (xanthine phosphoribosyltransferase); they were amplified and sequenced in both directions using *S. pneumoniae* primers. It was not possible to obtain amplicons using the primers designed for the amplification of the signal peptidase I (*spi*) locus and this was replaced with the mismatch repair protein (*hexB*), for which primers (*hexB-F*, 5′-ACCTATCTCTTTGCCCAG-3′, and *hexB-R*, 5′-CCTGAATACGTCGGAACATCTTT-3′) were designed from the *S. pneumoniae* R6 (NC 003098) genome sequence (GenBank AE007317). The same primers were used to amplify and sequence partial gene fragments in *S. pseudopneumoniae* DMZ 18670. *S. pneumoniae* allele sequences of *aroE*, *gdh*, *gki*, *recP* and *xpt* (the loci used here in common with those of *S. oralis*, *S. mitis* and *S. pseudopneumoniae*) were downloaded from http://spneumoniae.mlst.net.

### DNA extraction, PCR parameters and nucleotide sequencing.

Bacteria grown overnight were suspended in 100 μl sterile ultra-high quality (UHQ) water and heated on a heat block (Microtherm Camlab) for 10 min at 100 °C, centrifuged at 15 800 ***g*** (Microlite centrifuge, Thermo Electron Corporation) for 10 s and 20 μl of the supernatant containing genomic DNA was removed and placed in a fresh tube; the rest was discarded. The DNA was cleaned using MicroClean (Microzone) and resuspended in 20 μl sterile UHQ water with a DNA yield of approximately 50 ng μl^−1^. Amplification of the fragments of chosen loci was carried out using the primers described above. Each 25 μl reaction contained 22.5 μl reddyMix mastermix (ABgene, UK), 5 pmol forward primer, 5 pmol reverse primer and 1.5 μl DNA extract. The cycling parameters were: initial denaturation for 5 min at 96 °C followed by 30 cycles of 94 °C for 30 s, 49 °C for 30 s and 72 °C for 90 s. A final extension was carried out for 5 min at 72 °C. The PCR products were purified using Microclean (Microzone), rehydrated in the same volume of UHQ water and stored at –20 °C. The amplicons were sequenced in reactions containing 2 μl PCR product, 0.5 μl BigDye v3.0 (Applied Biosystems), 1.75 μl 5× solution buffer (Applied Biosystems), 1.75 μl sterile UHQ water and 4 μl primer (3 pmol). Both strands for each gene fragment were sequenced. The cycling protocol and cleaning of sequencing reaction products were as described in the manufacturer's protocols and sequencing was performed using an ABI 3730xl DNA Analyser (Applied Biosystems).

### Sequence data analysis

#### (i) *S. oralis* and *S. mitis*.

The sequences of the individual loci obtained from the isolates and type strains were assembled from the electropherograms using the Staden package (Staden, 1996) (http://staden.sourceforge.net/) and aligned, along with the *S. mitis* strain NCTC 12261 sequences with accession numbers for *aroE* (EU075875), *ddl* (EU075697), *gdh* (EU075731), *gki* (EU075767), *recP* (EU075804) and *xpt* (EU075662), using the BioEdit program (http://www.mbio.ncsu.edu/BioEdit/bioedit.html). The coding sequences used for the housekeeping gene fragments were read in-frame. Gene sequences that differed from each other by one or more polymorphisms were assigned an allele number in the order of discovery. Each unique allelic profile, as defined by the allele numbers of the seven loci (*aroE*, *ddl*, *gdh*, *gki*, *hexB*, *recP* and *xpt*), was assigned a sequence type (ST) number. The 113 oral isolates and the *S. oralis, S. mitis* and *S. pseudopneumoniae* type strains were considered together in the assignment of STs.

DnaSP version 4.0 (www.ub.es/dnasp/index.html) was used to calculate the G+C content of the sequences as well as the number of polymorphic sites, average number of parsimony informative sites and average non-synonymous/synonymous rate ratio (*d*_N_/*d*_S_) of *S. oralis* and *S. mitis* separately. The nucleotide diversity per site (*π*) and the average number of nucleotide differences per site (θ) were calculated for each gene of both species.

Evidence for recombination between STs was investigated using several approaches. Split decomposition trees were constructed with 1000 bootstrap replicates based on parsimony splits as implemented in SplitsTree 4.0 (http://www.splitstree.org). The resulting trees were analysed by the phi test (Bruen *et al.*, 2006), which determines the probability of observing the inferred nucleotide homoplasies under the assumption of no recombination. Evidence was also obtained by analysing all STs with the algorithms implemented in the RDP program (http://darwin.uvigo.es/rdp/rdp.html), in which significant (*P*<0.001) evidence of recombination had to be demonstrated with at least two tests for recombination to be considered statistically significant. Maximum-likelihood (ML) phylogenetic trees were constructed using paup version 4 beta 10 (Swofford, 2003). An ML tree for each of the seven genes was computed and compared using the Shimodaira–Hasegawa test, which determines if significant differences occur among the tree topologies (differences in log-likelihood, Δ−ln*L*). In the case of a clonal population, each gene tree would show the same phylogeny, and there should not be any significant differences in likelihood (Coffey *et al.*, 2006; Feil & Spratt, 2001). To assess the extent of congruence among the ML trees, randomization tests were performed (Holmes *et al.*, 1999), in which the Δ−ln*L* values for each of the seven genes were compared to the equivalent values computed for 200 random trees created from each gene. This analysis was carried out on all the data including *S. oralis* and *S. mitis* STs, and also on *S. oralis*-only STs and on selected STs, representative of clusters or discrete lineages identified within the *S. oralis* data.

Potential clonal complexes were identified from the *S. oralis* and *S. mitis* STs using eBURST (Feil *et al.*, 2004), and to confirm this output and to presumptively identify isolates on the basis of clustering with type strains we used ClonalFrame (Didelot & Falush, 2007). This program was also used to identify recombination events in each *S. oralis* locus. In order to compare our analyses with those reported by Bishop *et al.* (2009) and Kilian *et al.* (2008) we also constructed an NJ tree with mega version 4 (Tamura *et al.*, 2007) using the concatenated sequences.

#### (ii) Comparison of *S. pneumoniae*, *S. pseudopneumoniae*, *S. oralis* and *S. mitis*.

To compare the relatedness of the oral *S. oralis* and *S. mitis* isolates, *S. pseudopneumoniae* type strain and *S. pneumoniae,* the first 30 *S. pneumoniae* STs were downloaded from http://spneumoniae.mlst.net, the sequences of the five loci in common were concatenated and these were then analysed using ClonalFrame.

## RESULTS

### Genetic characterization

A total of 74 STs were identified from amongst the 113 clinical isolates and each of the three type strains was assigned a distinct ST (Supplementary Table S1). The allelic profile and isolate provenance data are publicly available at http://pubmlst.org/soralis/. The mean *d*_N_/*d*_S_ values for each of the genes were all <0.1, consistent with the seven loci being subject to stabilizing selection. The *S. mitis* gene sequences exhibited characteristics similar to those of the *S. oralis* genes (Table 1) and these characteristics of both species are similar to those of *S. pneumoniae* housekeeping genes (Whatmore *et al.*, 2000).

### Diversity among subjects

The majority of human subjects (54/57) harboured unique STs. ST-21, however, was isolated from three different subjects but one of these also harboured a unique ST (4). In those subjects from whom multiple isolates were analysed, the majority (13/19) were found to harbour more than one ST. In one subject, eight isolates were analysed and these were typed as six distinct STs (9, 17, 13, 24, 31 and 37) while from another subject eight isolates were found to be identical (ST-6). All isolates with the same ST isolated from the same individual had the same REP-PCR pattern and all isolates from the same subject with different STs had a different REP-PCR pattern, except for one subject in whom the same REP-PCR pattern was associated with different STs. ST-21 was isolated from three different subjects and each ST-21 isolate exhibited a different REP-PCR pattern. Thus, overall, there was a high degree of concordance between the assignment of an ST and the REP-PCR.

### Interrelatedness of sequence types

**Fig. 1. f1:**
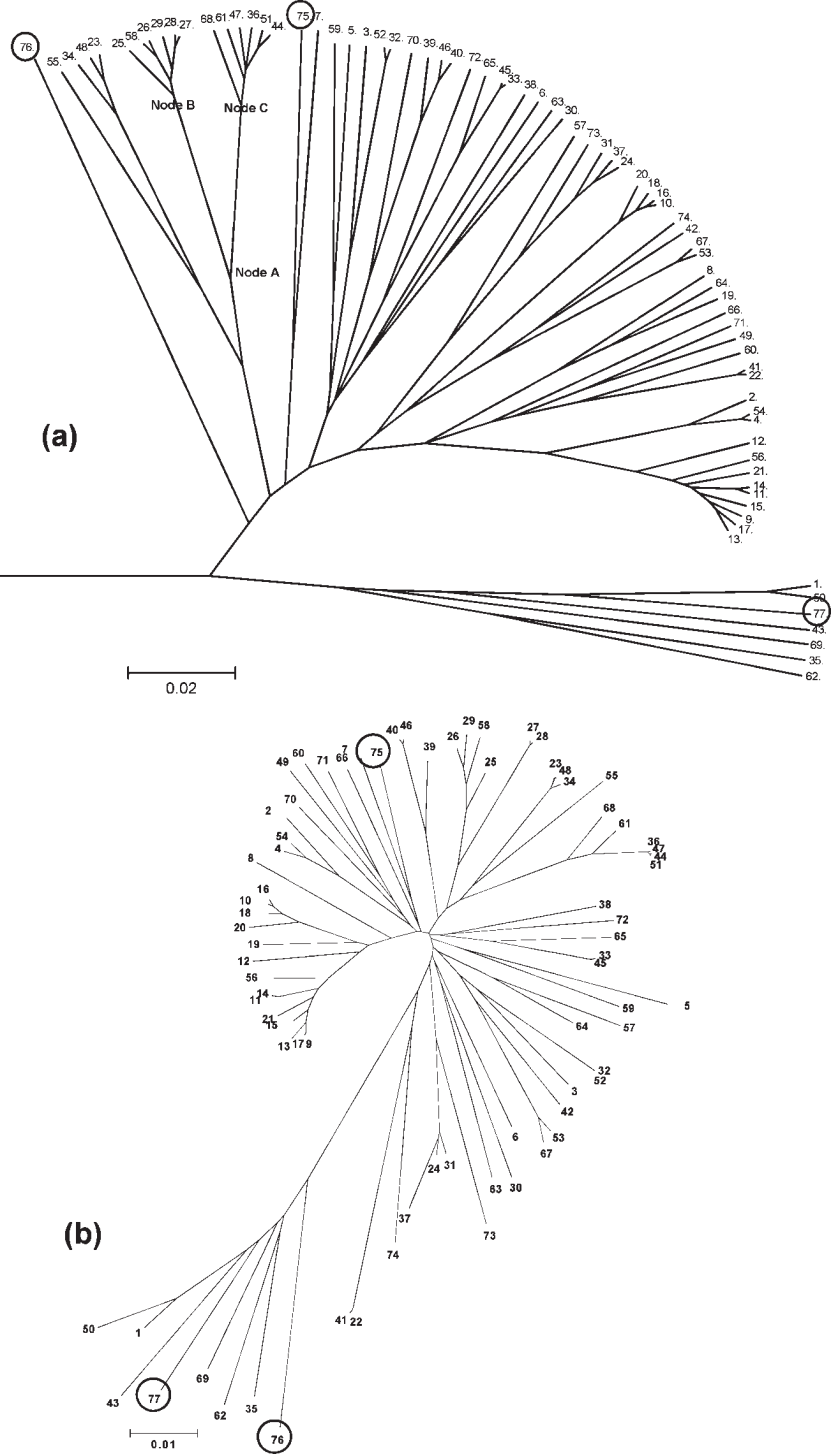
(a) Radial phylogenetic tree constructed with the ClonalFrame program, displaying the clonal relationship between the STs of the *S. oralis/mitis* population at seven loci. The dendrogram was constructed from the combination of six ClonalFrame runs (with a cut-off value of 0.5, as a majority rule consensus; scale is coalescent units). (b) NJ tree constructed in mega v4.0, using the MLST concatenated sequences, and showing the relationship between the STs. ST-75, ST-76 and ST-77 (circled in both trees) are the STs for the *S. oralis*, *S. pseudopneumoniae* and *S. mitis* type strains, respectively.

**Fig. 2. f2:**
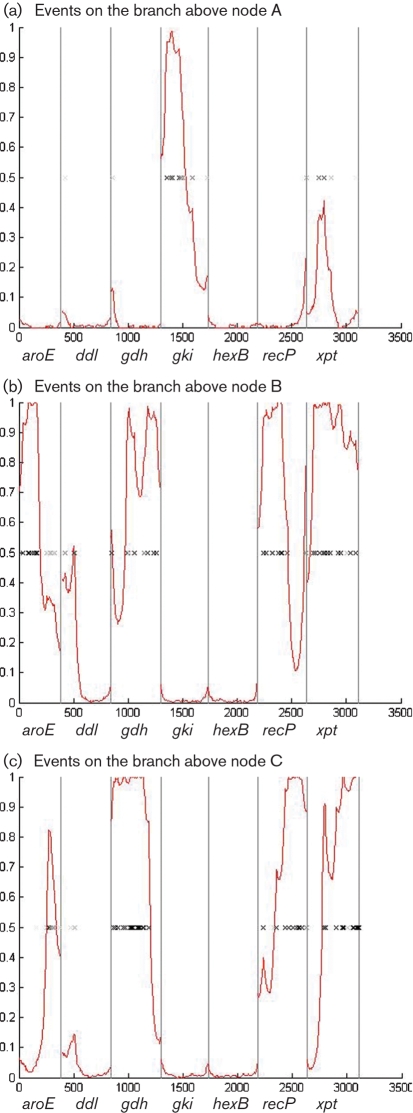
Graphical representations of the nucleotide differences (marked by crosses) in the concatenated sequences of *aroE, ddl, gdh, gki, hexB, recP* and *xpt* (as shown on the *x*-axis), above nodes A, B and C indicated in Fig. 1. The red lines indicate the probability that a nucleotide change has resulted from recombination, based on the clustering of changes.

Analysis with eBURST identified three putative complexes with founders (STs 15, 26 and 61) and eight groups consisting of pairs of STs exhibiting some relationship to each other. ClonalFrame and neighbour-joining (NJ) tree analysis of the concatenated sequences of the 113 clinical isolates identified two distinct clusters (Fig. 1). One of these, which contained the *S. oralis* type strain, consisted of 107 isolates that were presumed to be *S. oralis*. In the ClonalFrame analysis the remaining six isolates clustered with the *S. mitis* type strain and were presumed to be *S. mitis*, and none of the clinical isolates clustered with the type strain of *S. pseudopneumonia*e, which formed a distinct lineage. In the NJ tree, however, the type strain of *S. pseudopneumonia*e was more closely related to the *S. mitis* cluster. More detailed relationships were apparent within the ClonalFrame tree, which showed evidence of a clonal complex structure for *S. oralis*, with STs forming clusters at the end of long branches and other STs as members of more diffuse clusters. The mutation and recombination events that introduced polymorphisms at these cluster nodes were investigated further. Of the three nodes (A, B and C) of the ClonalFrame tree indicated in Fig. 1(a), the bifurcation at node A is characterized by a likely recombination event at the *gki* locus and either a recombination or multiple mutations at the *xpt* locus (Fig. 2). Further diversification of the two resulting clusters, above nodes B and C, was due to modifications in *aroE*, *gdh, recP* and *xpt*, with the pattern of nucleotide changes consistent with recombination.

**Fig. 3. f3:**
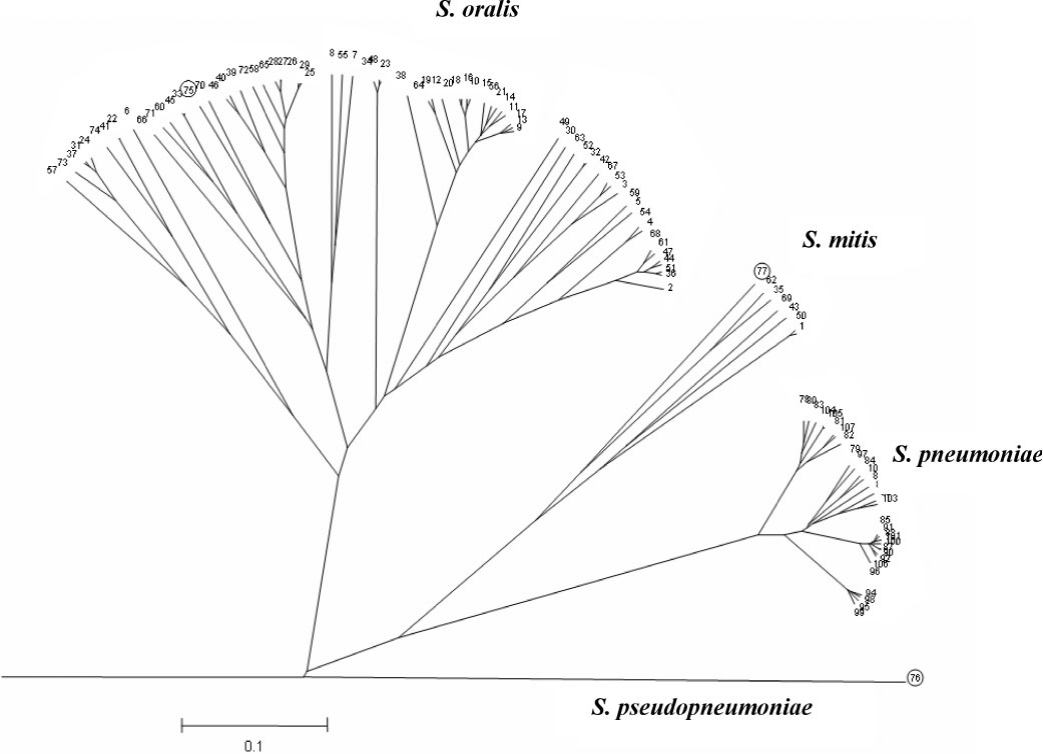
Radial phylogenetic tree constructed with the ClonalFrame program for the five loci of the *S. oralis* and *S. mitis* STs and the 30 *S. pneumoniae* STs from http://spneumoniae.mlst.net/. ST-75, ST-76 and ST-77 (circled) are the STs for the *S. oralis*, *S. pseudopneumoniae* and *S. mitis* type strains, respectively. The dendrogram was constructed from the combination of six ClonalFrame runs (with a cut-off value of 0.5, as a majority-rule consensus). Scale is coalescent units.

A ClonalFrame comparison using five genes (*aroE, gdh, gki, recP* and *xpt*) common in this analysis to all STs and to the *S. pneumoniae* MLST scheme showed that the type strain of *S. pseudopneumoniae* was represented by a separate lineage (Fig. 3) and not part of the *S. mitis* cluster as reported by (Kilian *et al.*, 2008). The data for *ddl* alleles were excluded from this analysis as *ddl* sequence polymorphism was shown to be linked to acquisition of genes for *pbpA* in *S. pneumoniae*, meaning that environmental factors may drive *ddl* gene diversity (Enright & Spratt, 1998; Chi *et al.*, 2007).

### Recombination within *S. oralis*

**Fig. 4. f4:**
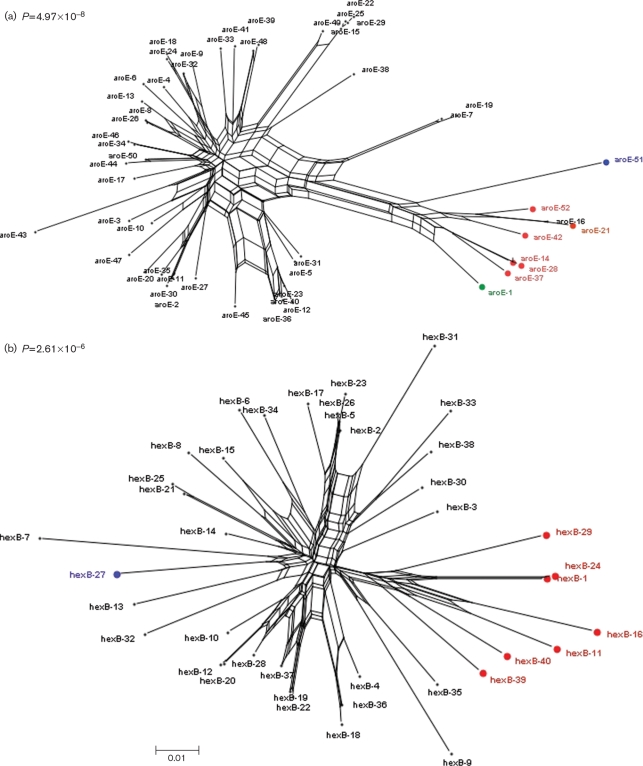
NeighborNet graphs of STs for the *aroE* (a) and *hexB* (b) loci, constructed in SplitsTree v4.0. The black-labelled STs are *S. oralis* loci, the blue label denotes *S. pseudopneumoniae*, the red labels denote the *S. mitis* loci and the green label corresponds to the one allele shared by both *S. oralis* and *S. mitis*.

Split decomposition analysis of each locus of the *S. oralis* and *S. mitis* STs produced complex networks consistent with recombination. Comparison of the NeighborNet graph for *aroE* (Fig. 4a[Fig f4]) showed that allele aroE-16, found in ST-37 and identified as *S. oralis*, clustered with the *S. mitis* alleles. Further, allele aroE-1, which also clustered with *S. mitis* alleles, was present in STs of both *S. oralis* (ST-64) and *S. mitis* (ST-1). *S. pseudopneumoniae* alleles clustered with *S. mitis* for all loci (Fig. 4 and Supplementary Fig. S1) with the exception of *hexB* (Fig. 4b[Fig f4]) in which the *S. pseudopneumoniae* allele was situated within the *S. oralis* cluster.

**Fig. 5. f5:**
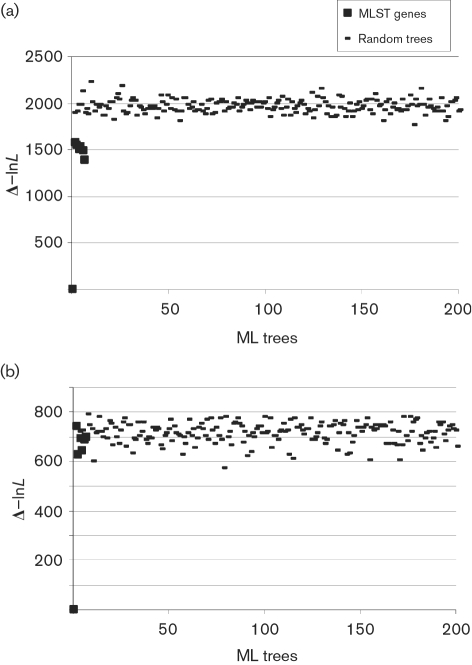
Congruence of the *aroE* ML tree with trees generated from other MLST loci and 200 random trees for *S. oralis* STs (a) and for selected STs (34, 26, 47, 75, 7, 59, 5, 3, 32, 70, 40, 72, 65, 33, 38, 6, 63, 30, 57, 73, 37, 18, 74, 42, 53, 8, 64, 19, 66, 71, 49, 60, 41, 54, 12, 11) as single representatives of the individual clusters identified in the ClonalFrame analysis (Fig. 1) (b). The same pattern was observed with the other loci (data not shown).

The Shimodaira–Hasegawa test for congruence demonstrated that all ML trees generated for individual MLST loci were significantly incongruent with each other (Supplementary Table S2); however, comparison of the Δ−ln*L* values for individual ML trees indicated that these were consistently more similar to each other than randomly generated trees (Fig. 5a[Fig f5]). To test if the small level of congruence was the result of a clonal complex structure, the test was repeated using only single STs, representative of the individual clusters found in the ClonalFrame analysis (Fig. 1a[Fig f1]). In this case, the congruence between the ML trees constructed for individual loci was not significantly different from random trees (Fig. 5b[Fig f5]).

Significant recombination was detected at the *aroE* (*P*=4.91×10^−5^), *hexB* (*P*=2.35×10^−5^), *ddl* (*P*=2.705×10^−5^) and *xpt* (*P*=8.28×10^−3^) loci by the phi test. In order to identify other possible recombination events, all data were analysed using the RDP suite of programs. No significant evidence for intra-species recombination within *S. oralis* for the *ddl*, *gki* or *hexB* loci was detected. For the remaining loci there was significant evidence for recombination, but in all cases the total number of unique recombination events was limited. Thus for the *xpt* locus three recombination events were identified: in all cases the minor parent was xpt-22 and the major parent xpt-35, with daughters identified as xpt-15, xpt-6 and xpt-4. In *ddl* only a single recombination event was identified, with ddl-34 as the minor parent, ddl-34 as the major parent and ddl-2 as the daughter. Six recombination events were identified in *recP*, with recP-10 as the minor parent, recP-16 as the major parent and repP-4, -5, -6, -15, -18 and -21 as the daughters. The situation with *gdh* was more complicated. Two unique recombination events were observed. In the first, gdh-29 and gdh-21 were the minor and major parents respectively, and gdh-6 and gdh-25 the daughters. In the second event gdh-6 and gdh-30 were the minor and major parents respectively, and gdh-15 and gdh-20 the daughters. However, in alleles gdh-8 and gdh-37 both recombination events were identified. Thus these analyses provided statistical support for intra-species recombination within *S. oralis*. The small number of recombination events evident in these four alleles was subsequently augmented with varied mutational or undetected recombinational events resulting in further allelic diversity and the consequent generation of new STs. There was no concordance between the statistical support for recombination found with the phi-test used within Splitstree and the evidence provided by the RDP analysis.

## DISCUSSION

The development of an MLST scheme for *S. oralis* has demonstrated that the organism has a diverse population undergoing inter- and intra-species recombination, and has further resolved the relationship of this bacterium to related bacteria. It provides a tool for the further analysis of this bacterium, enabling the benefits of portability and comparability combined with data sharing via the web inherent in MLST to be extended to this organism. The high level of inter- and intra-individual heterogeneity in *S. oralis* and *S. mitis* isolates has been reported previously (Alam *et al.*, 2000; Hohwy *et al.*, 2001) using different strain comparison techniques and it mirrors the great inter-individual heterogeneity found in other commensal oral bacteria, including *Streptococcus mutans* (Nakano *et al.*, 2007), *Porphyromonas gingivalis* (Enersen *et al.*, 2006) and *Fusobacterium nucleatum* (Haraldsson *et al.*, 2004).

The clustering of isolates into named species groups on the basis of the concatenated MLST loci was similar, but not identical, to that reported previously. Specifically, the ClonalFrame analysis (Fig. 1a[Fig f1]) did not support the clustering of *S. pseudopneumoniae* with *S. mitis*, suggested by the NJ analysis here (Fig. 1b[Fig f1]) and elsewhere (Kilian *et al.*, 2008; Bishop *et al.*, 2009). Analysis of the individual loci (Fig. 4 and Supplementary Fig. S1) demonstrated that these inconsistencies were due to the presence in *S. pseudopneumoniae* of alleles at some loci clustering with the loci from *S. oralis*-derived sequences, while the alleles from other loci clustered with sequences characteristic of *S. mitis*. This suggests that this organism may represent a hybrid generated by interspecies gene exchange, as has been reported in other bacteria, notably *Campylobacter coli* (Sheppard *et al.*, 2008). Given the limited number of loci and isolates of *S. pseudopneumoniae* investigated to date here and elsewhere, the status of isolates belonging to this species must remain uncertain, although its candidacy as a hybrid bacterium warrants further investigation by genome-wide analysis of further isolates.

The *S. oralis* isolates examined were highly diverse, with very few STs identified more than once and few groups identified with the eBURST algorithm. This could be due either to an essentially non-clonal population structure, such as that seen in the gastric pathogen *Helicobacter pylori* (Falush *et al.*, 2001), or to a poorly sampled population with a clonal complex structure, the population structure seen in other related naturally transformable bacteria that have been studied more extensively such as *S. pneumoniae* (Feil *et al.*, 2004). The ML analysis of all STs compared to that including only unrelated STs, which demonstrated less congruence (Fig. 5), combined with the structuring in the ClonalFrame analyses (Figs 1 and 3), suggests a clonal complex structure. The levels of sequence diversity among the isolates investigated here, and the close relationship of *S. oralis* to other bacteria with a clonal complex population structure, further supports the contention that these isolates represent a small subset of a highly diverse recombining population structured into clonal complexes, although final resolution of this issue will depend on analysis of an expanding database of MLST data for *S. oralis*.

A particular advantage of MLST is that it is possible to compare data from studies conducted at different times and in different places. Although it was not possible to use all of the loci employed in the *S. pneumoniae* MLST scheme for *S. oralis*, it was possible to conduct a five-locus ClonalFrame analysis using the MLST loci that the two schemes have in common (*aroE*, *gdh*, *gki*, *recP* and *xpt*) (Fig. 3). The data for the *S. pneumoniae ddl* locus were excluded from this analysis as *ddl* sequence polymorphism is linked to acquisition of genes for *pbpA* (Enright & Spratt, 1998; Chi *et al.*, 2007). This analysis showed that from the current data it is not possible to reconstruct with certainty the evolutionary events that have led to the emergence of the three present-day populations from a common ancestral population and that it is premature, as well as genealogically unsound, to assign any of the present-day populations of *S. oralis*, *S. mitis* or *S. pneumoniae* as an ancestral group (Kilian *et al.*, 2008). Rather, it is more accurate to represent the three species examined here as three diverse modern populations which have all emerged from a single ancestral population. As mentioned previously, the possibility that the *S. pseudopneumoniae* isolate examined is a hybrid organism, containing genetic material from more than one parental population, makes its position in the genealogy of oral streptococci uncertain until further isolates and loci have been examined. These data demonstrate the limitations of analyses based on distance-based algorithms, such as NJ trees, that cannot account for the effects of recombination.

The oral streptococci are highly diverse and competent for transformation with exogenous DNA; however, although recombination events do occur between species, they are relatively rare, with the species groups representing coherent, distinct populations. The well-known species *S. mitis*, *S. pneumoniae* and *S. oralis* are therefore valid taxonomic units although there is extensive genetic variability within them. Within *S. oralis*, there is evidence of recombination but also a possible clonal complex structure. The mechanism whereby this structuring is maintained is yet to be elucidated but a number of possible mechanisms can be invoked (Fraser *et al.*, 2009). Understanding the interrelationships of distinct groups and especially the relationships of genotypes to colonization and disease phenotypes is only possible once well-characterized isolate collections representative of natural populations have been assembled. The MLST scheme and its associated database (http://pubmlst.org/soralis) reported here represents a first step in this process.

## Figures and Tables

**Table 1. t1:** Characteristics of loci of the *S. oralis* (*n*=69) and *S. mitis* (*n*=7) sequence types assigned to the clinical isolates and type strains *π*, Nucleotide diversity per site; θ, average number of nucleotide differences per site.

**Locus**	**Species**	**Alleles**	**G+C**	**No. of polymorphic sites**	**No. of parsimony-informative sites**	***π***	**θ**	***d*_N_/*d*_S_**
*aroE* (398 bp)	*S. oralis*	43	0.442	110	81	0.0589	0.0830	0.089
	*S. mitis*	7		30	30	0.0532	0.0533	0.098
*ddl* (441 bp)	*S. oralis*	34	0.447	108	72	0.0564	0.0721	0.057
	*S. mitis*	6		58	40	0.0332	0.0620	0.038
*gdh* (459 bp)	*S. oralis*	31	0.436	111	84	0.0516	0.0698	0.013
	*S. mitis*	6		37	15	0.0745	0.0347	0.029
*gki* (432 bp)	*S. oralis*	28	0.471	78	54	0.0403	0.0545	0.024
	*S. mitis*	6		49	24	0.0488	0.0501	0.009
*hexB* (455 bp)	*S. oralis*	31	0.475	127	83	0.0609	0.0798	0.017
	*S. mitis*	7		75	48	0.0745	0.0727	0.031
*recP* (448 bp)	*S. oralis*	24	0.449	94	62	0.0402	0.0646	0.006
	*S. mitis*	6		39	19	0.0390	0.0383	0.030
*xpt* (488 bp)	*S. oralis*	27	0.445	119	82	0.0622	0.0787	0.075
	*S. mitis*	6		56	19	0.0442	0.0494	0.055

## Abbreviations

- ML, maximum likelihood
- MLST, multilocus sequence typing
- NJ, neighbour joining
- REP-PCR, repetitive extragenic palindromic-PCR
- ST, sequence type
- UHQ, ultra-high quality
